# Identification and characterisation of LEAP2 from Chinese spiny frogs (*Quasipaa spinosa*) with antimicrobial and macrophage activation properties

**DOI:** 10.1186/s12917-025-04617-y

**Published:** 2025-03-13

**Authors:** Ping Ying, Xin-Yi Qian, Zi-Xuan Wang, Jia-Le Wu, Jia-Yin Huang, Zi-Yi Ren, Jie Chen

**Affiliations:** 1 Zhejiang Lishui Service Platform for Technological Innovations in Traditional Chinese Medicine Industry, Lishui University, Lishui, 323000 China; 2 College of Ecology, Lishui University, Lishui, 323000 China; 3 College of Animal Sciences, Zhejiang University, Hangzhou, 310058 China

**Keywords:** Chinese spiny frog, LEAP2, Gene expression, Antimicrobial activity, Immunomodulatory activity

## Abstract

**Background:**

The liver-expressed antimicrobial peptide 2 (LEAP2) family is an important group of antimicrobial peptides (AMPs) involved in vertebrate defence against bacterial infections. However, research on LEAP2 in amphibians is still in its infancy.

**Results:**

This study aimed to explore the role of LEAP2 in the Chinese spiny frog (*Quasipaa spinosa*). The cDNA of the *LEAP2* gene (*QsLEAP2*) was cloned from a Chinese spiny frog. The QsLEAP2 protein comprises a signal peptide, a prodomain, and a mature peptide. Sequence analysis indicated that QsLEAP2 is a member of the amphibian LEAP2 cluster and closely related to the LEAP2 of the African clawed frog (*Xenopus laevis*). Expression of *QsLEAP2* was detected in various tissues, with the liver exhibiting the highest expression. Following infection with *Aeromonas hydrophila*, *QsLEAP2* expression was significantly upregulated in the spleen, lungs, kidneys, liver, and gut. The synthetic mature peptide QsLEAP2 exhibited selective antimicrobial activity against several bacterial strains in vitro. It disrupted bacterial membrane integrity and hydrolysed bacterial genomic DNA, exhibiting bactericidal effects on specific bacterial species. Furthermore, QsLEAP2 induced chemotaxis in RAW264.7 murine leukemic monocytes/macrophages, enhancing their phagocytic activity and respiratory bursts. Docking simulations revealed an interaction between QsLEAP2 and QsMOSPD2.

**Conclusions:**

These findings provide new insights into the role of LEAP2 in the amphibian immune system.

**Supplementary Information:**

The online version contains supplementary material available at 10.1186/s12917-025-04617-y.

## Background

Antimicrobial peptides (AMPs) have emerged as promising treatments for antimicrobial resistance, drawing significant scientific interest. Due to the growing issue of antibiotic resistance, AMPs are a key research focus due to their broad antimicrobial spectrum and low resistance propensity [1]. These small peptides, composed of positively charged amino acids, bind to bacterial cell membranes, causing cell lysis [2]. AMPs are effective against both Gram-positive and Gram-negative bacteria, and also inhibit fungi and viruses [3]. Additionally, AMPs have immunomodulatory functions, playing a significant role in host immune defence. They can stimulate immune cells, facilitate cytokine release [4], and regulate inflammatory responses, contributing to immune homeostasis [5]. In some cases, the immunomodulatory effects of AMPs may outweigh their antimicrobial activities [4].

Liver-expressed antimicrobial peptide 2 (LEAP2) is an important AMP found in vertebrates, playing a central role in the innate immune system by protecting against bacterial infections [6]. LEAP2 contains four highly conserved cysteine residues that form two disulphide bonds, which constitute its core structure [7]. Additionally, LEAP2 acts as an endogenous antagonist of the growth hormone secretagogue receptor (GHSR), regulating appetite, energy metabolism, and growth hormone signalling [8]. Moreover, LEAP2 influences immune system regulation by activating immune cells and promoting cytokine expression, enhancing the body’s defence against pathogens [9]. LEAP2 administration reportedly enhances survival rates in infected animals and reduces bacterial load, according to the findings of relevant study [10]. In conclusion, LEAP2 is a multifunctional AMP involved in infection defence and the regulation of energy metabolism and immune responses, making it promising for drug development [1].

Amphibian LEAP2 has been reported in the Chong’an moustache toad (*Leptobrachium liui*) [11] and the African clawed frog (*Xenopus laevis*) [12]. In African-clawed frogs, LEAP2 plays a significant role in embryonic development and cellular signalling, with high expression in endoderm-derived tissues regulated by fibroblast growth factor (FGF) and activin [12]. The antibacterial activity and mechanism of LEAP2 have been studied in Chong’an moustache toads [11]. The Chinese spiny frog (*Quasipaa spinosa*), predominantly found in Southeast Asia, has commercial and nutritional value, but its viability is threatened by bacterial infections [13]. In this study, we aimed at identifying and characterising a LEAP2 homologue (QsLEAP2) from the Chinese spiny frog and investigate its dual antimicrobial and immunomodulatory functions. Our objectives included the molecular cloning and structural analysis of QsLEAP2, profiling its tissue-specific and infection-inducible expression patterns, and evaluating its in vitro antibacterial activity against pathogenic bacteria. Furthermore, we explored the immunoregulatory potential of QsLEAP2 by assessing macrophage chemotaxis, phagocytosis, and respiratory burst responses. The methods we applied encompass transcriptome-based cloning, qPCR, synthetic peptide testing, and in silico docking. This comprehensive approach provides foundational insights into the multifunctional role of LEAP2 in amphibian immunity, with implications for developing novel antimicrobial therapies.

## Results

### Molecular characterisation of QsLEAP2

*QsLEAP2* has been deposited in the GenBank database and assigned the accession number PQ528275. The ORF of *QsLEAP2* consisted of 288 nucleotides and encoded 95 amino acids. The QsLEAP2 protein comprised three distinct sections: a signal peptide, a prodomain, and a mature peptide. The mature peptide consisted of 41 amino acids with a relative molecular mass of 4.80 kDa and an isoelectric point of 9.58. The multiple sequence alignment of vertebrate LEAP2 results suggest that the mature peptide was more conserved than the signal peptide and propeptide. Four conserved cysteine residues within the mature peptide formed two pairs of disulfide bonds (Fig. . 1A). The 3D structural modelling predictions indicate that the mature peptide of the QsLEAP2 protein comprised α-helices and β-sheets. The mature QsLEAP2 peptide contained four distinctive cysteine residues that participate in the formation of two disulfide bonds, which may result in the generation of a hydrophilic cluster and the exposure of cationic residues on the surface (Fig. . 1B). Phylogenetic tree analysis revealed that amphibian LEAP2 formed a distinct cluster separate from those of mammals, birds, reptiles, and fish. QsLEAP2 shares its closest relationship with the African-clawed frog (*Xenopus laevis*) (Fig. . 2).

**Fig. 1:**
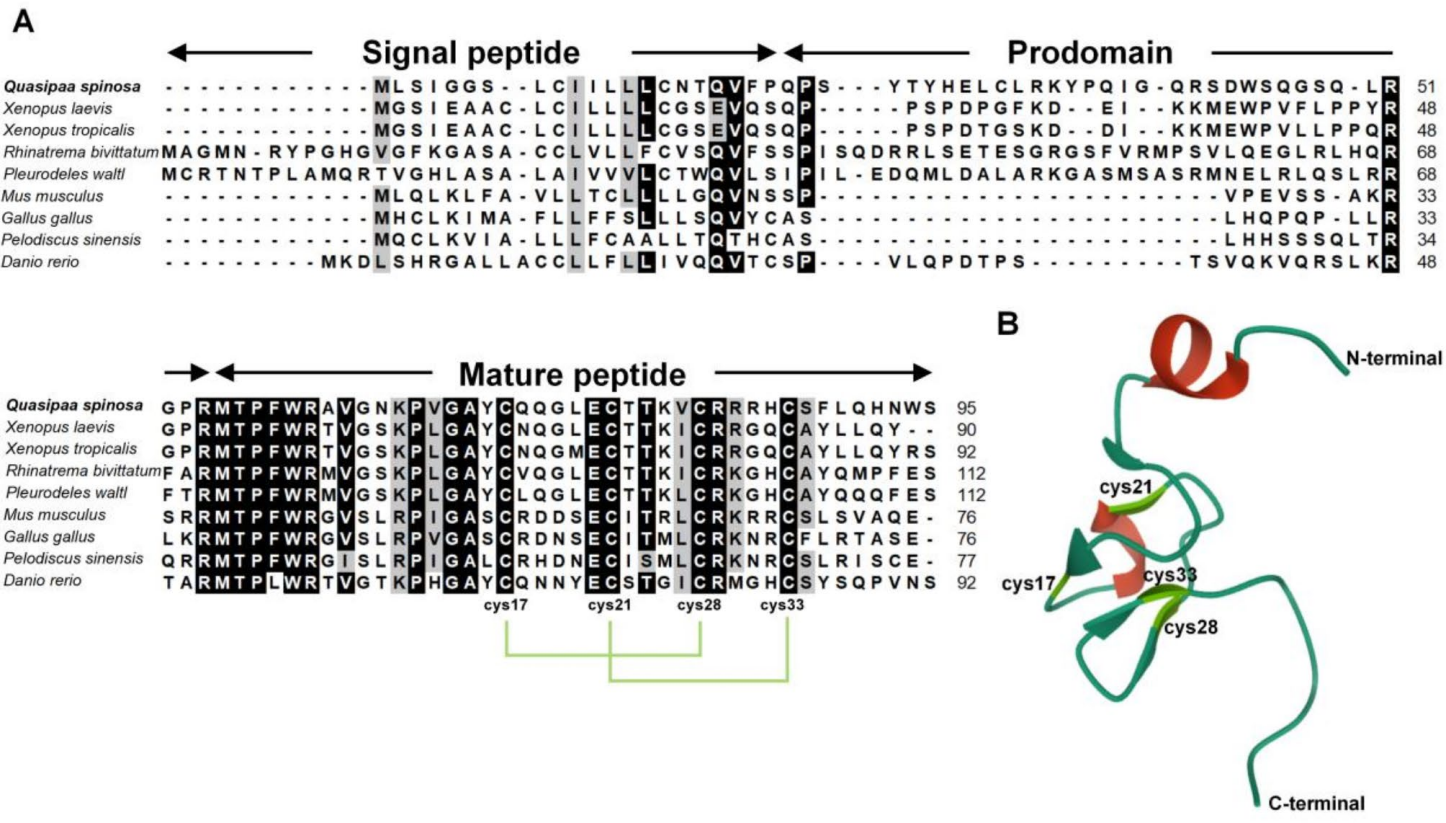
Multiple sequence alignment and three-dimensional structure prediction of QsLEAP2. (**A**) Multiple sequence alignment of the QsLEAP2 protein and its homologues. The shading threshold was set at 70%; similar residues are highlighted in grey, identical residues are in black, and alignment gaps are denoted by “-”. (**B**) AlphaFold2 was used to model the three-dimensional structure of QsLEAP2

**Fig. 2:**
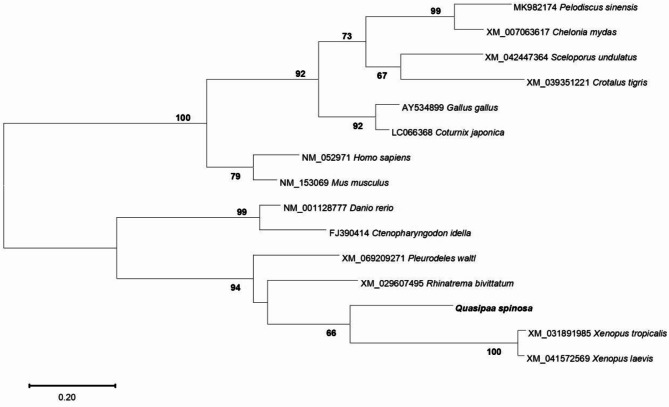
Phylogenetic analysis of LEAP2 amino acid sequences was performed with MEGA X using the neighbour-joining method. Branch point values show the percentage of trees with that grouping from a bootstrap analysis (1000 replicates; shown only if over 60%). The scale bar represents substitutions per base

### Constitutive and infection-inducible expression of QsLEAP2

*QsLEAP2* was expressed ubiquitously across all examined tissues, with significantly elevated expression in immune-related tissues, such as the liver, kidneys, and spleen. The highest expression was observed in the liver where it was 104.16-fold higher than that in muscle (Fig. . 3A). Upon infection with *Aeromonas hydrophila*, *QsLEAP2* exhibited a sustained upregulation in the gut, spleen, and kidneys, with respective 6.66-fold, 3.46-fold, and 2.62-fold increases (Fig. . 3C-E). Conversely, in the liver and lungs, *QsLEAP2* was initially upregulated, then downregulated, with peak upregulations reaching 1.91-fold and 4.27-fold, respectively (Fig. . 3B, F).

**Fig. 3:**
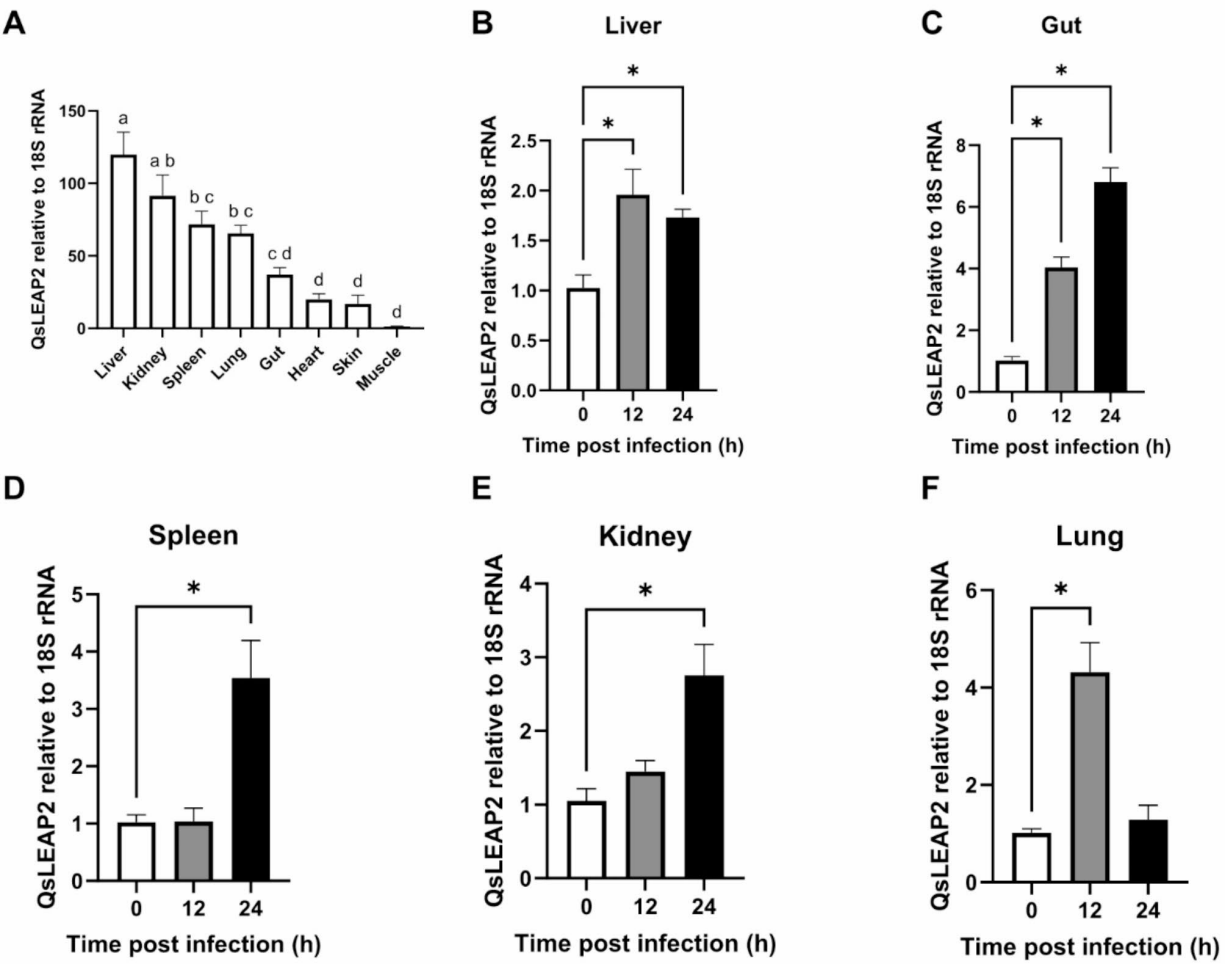
Expression of *QsLEAP2*: (**A**) Tissue-specific expression, with significant differences indicated by letters (one-way ANOVA, *P* < 0.05). (**B**-**F**) Changes in QsLEAP2 expression post-*Aeromonas hydrophila* infection, normalised to *Qs18S rRNA*. Data are mean ± SEM, analysed by one-way ANOVA, *n* = 4, *P < 0.05

### In vitro antibacterial activity of QsLEAP2

The antibacterial activity of the QsLEAP2 mature peptide was evaluated by determining its minimal inhibitory concentration (MIC) against a range of bacteria. The QsLEAP2 mature peptide exhibited the strongest antibacterial activity against *Shigella flexneri* and *Vibrio alginolyticus* (MIC = 3.125 µg/mL). The next strongest activity was observed against *Salmonella enterica* (MIC = 6.25 µg/mL) and *Staphylococcus aureus* (MIC = 50 µg/mL). The weakest antibacterial activity was observed against *Staphylococcus warneri* (MIC = 100 µg/mL). However, no antibacterial activity was observed against *Listeria monocytogenes*, *Staphylococcus saprophyticus*, *Escherichia coli*, *Pseudomonas aeruginosa*, *A. hydrophila*, and *Proteus mirabilis* (Table 1).

**Table 1:** Antibacterial activity of synthetic QsLEAP2 mature peptide

Bacteria	Isolate/strain	MIC (µg/mL)
Gram-negative		
*Shigella flexneri*	ATCC12022	3.125
*Vibrio alginolyticus*	ATCC17749	3.125
*Salmonella enterica*	ATCC13076	6.25
*Proteus mirabilis*	ATCC25933	-
*Aeromonas hydrophila*	ATCC7966	-
*Pseudomonas aeruginosa*	ATCC27853	-
*Escherichia coli*	K12	-
Gram-positive		
*Staphylococcus aureus*	ATCC6538	50
*Staphylococcus warneri*	ATCC49454	100
*Staphylococcus saprophyticus*	ATCC49907	-
*Listeria monocytogenes*	ATCC19115	-

The hyphen symbol (-) denotes the absence of inhibitory activity at the maximum tested concentration of 100 µg/mL

### Impact of QsLEAP2 on Shigella flexneri cell membrane integrity and genomic DNA

**Fig. 4:**
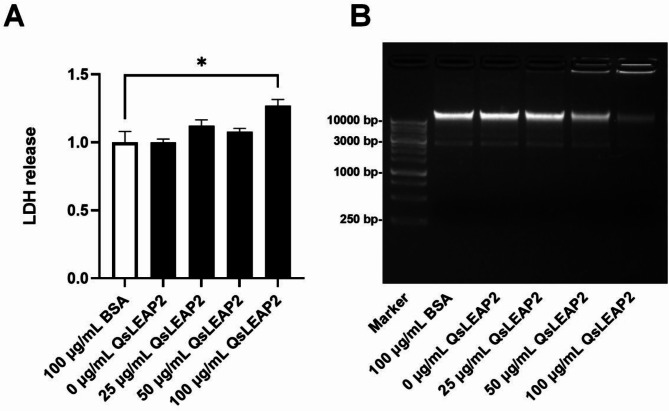
Impact of QsLEAP2 on *Shigella flexneri* cell membrane and genomic DNA. (**A**) QsLEAP2 affects *S. flexneri* cell membrane integrity, with LDH release shown as fold-change relative to a negative control (BSA, set at 1). Data are mean ± SEM from four experiments, analysed by one-way ANOVA (*P < 0.05). (**B**) Hydrolytic activity of QsLEAP2 on bacterial genomic DNA, with BSA as a negative control; one of four independent experiments is presented. Marker: 1 Kb DNA Marker P (Sangon; lot: B600022)

The lactate dehydrogenase (LDH) release assay showed that 100 µg/mL of QsLEAP2 could effectively promote the release of LDH, indicating that at this concentration, QsLEAP2 can disrupt the cell membrane of *S. flexneri* (Fig. 4A). Following the incubation of genomic DNA from *S. flexneri* with QsLEAP2 and subsequent detection of the genomic DNA bands via agarose gel electrophoresis, a reduction in band intensity was observed at concentrations of 50 µg/mL and 100 µg/mL of QsLEAP2. This suggests that QsLEAP2 efficiently degraded the genomic DNA of *S. flexneri* at these two concentrations (Fig. 4B).

### Effect of QsLEAP2 on RAW264.7 cell chemotaxis

**Fig. 5:**
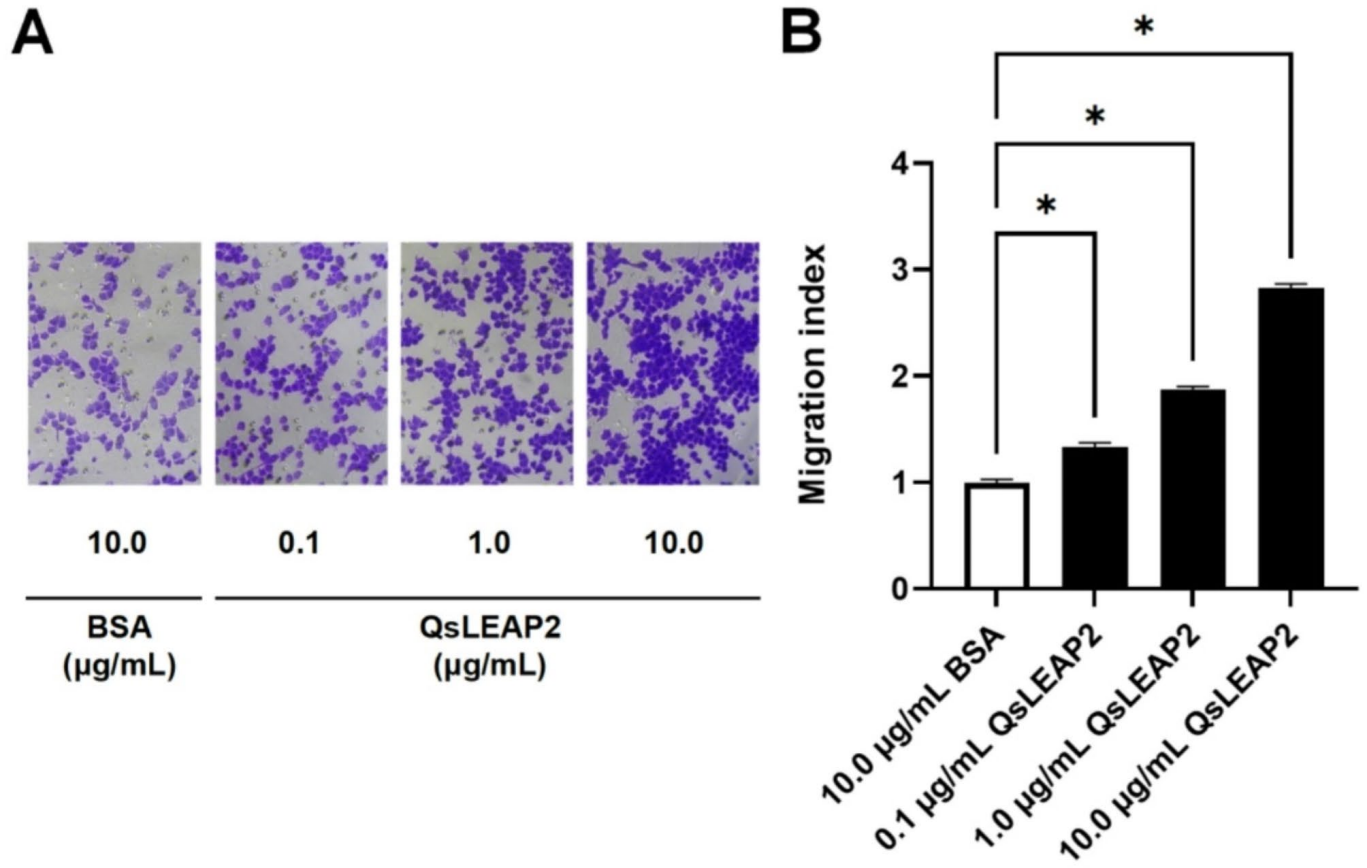
The impact of QsLEAP2 on RAW264.7 cell chemotaxis. Results are presented as mean ± SEM; *n* = 4; *P < 0.05

The chemotactic effect of QsLEAP2 on RAW264.7 cells was identified using a transwell chamber. The results demonstrated that RAW264.7 cells exhibited notable chemotactic responses to QsLEAP2 at concentrations of 0.1, 1.0, and 10.0 µg/mL. In comparison to the BSA group, the migration of cells in the 10.0 µg/mL QsLEAP2 group increased by 2.83-fold (Fig. 5).

### Effect of QsLEAP2 on RAW264.7 cell phagocytocis

**Fig. 6:**
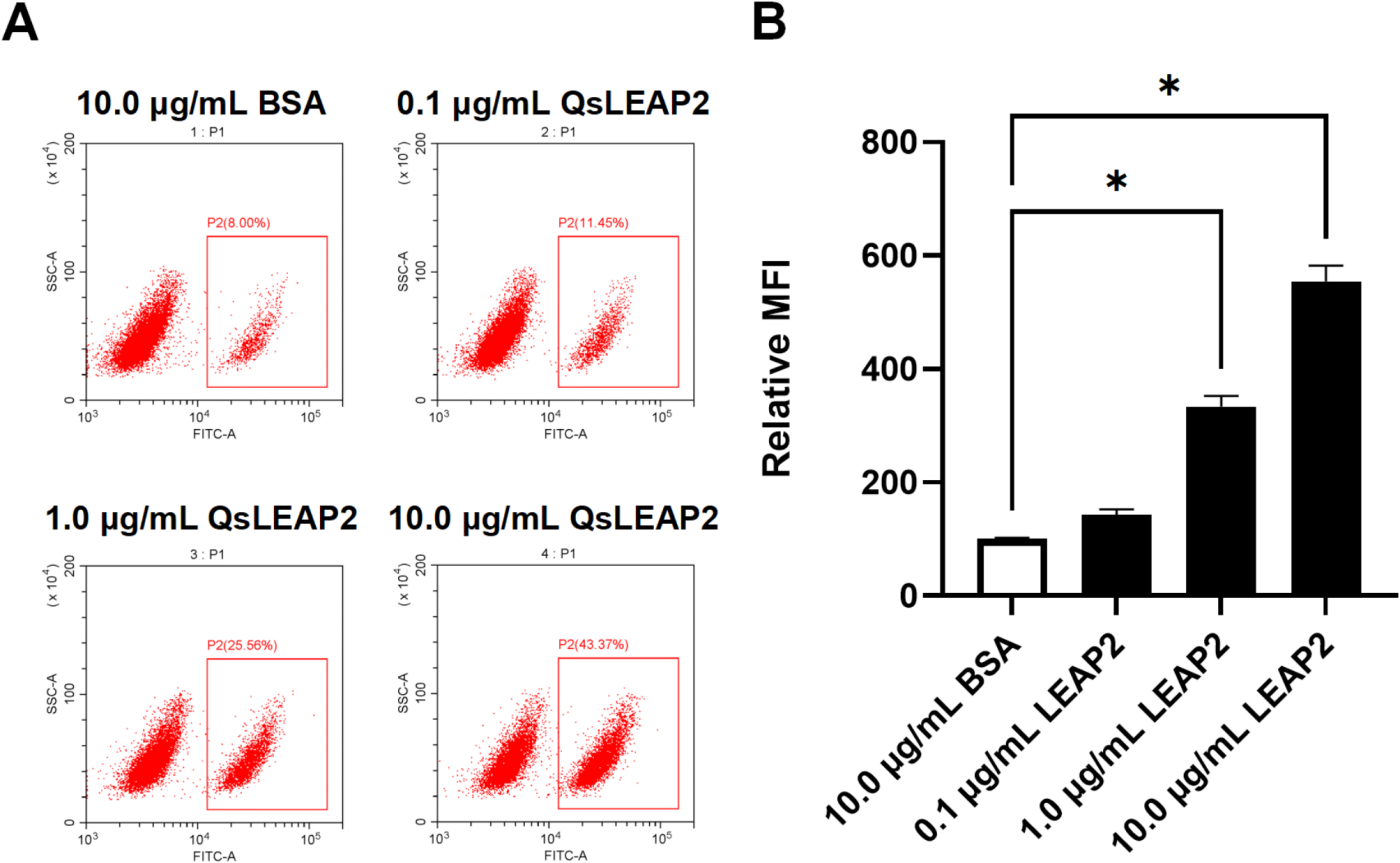
The effect of QsLEAP2 on the phagocytic activity of RAW264.7 cells. The mean fluorescence intensity (MFI) was reported as a fold-change in comparison to the BSA-treated control group, which was arbitrarily set to a value of 100. Results are depicted as the mean ± SEM; *n* = 4; *P < 0.05

The impact of QsLEAP2 on the phagocytic activity of RAW264.7 cells was evaluated using flow cytometry. The results showed that as the concentration of QsLEAP2 increased, the phagocytic uptake of FITC-dextran by RAW264.7 cells also rose. Treatment with 10.0 µg/mL QsLEAP2 resulted in a 2.54-fold increase in the phagocytic uptake of FITC-dextran by RAW264.7 cells, in comparison to the BSA treatment (Fig. 6)

### Effect of QsLEAP2 on RAW264.7 cell respiratory burst

**Fig. 7:**
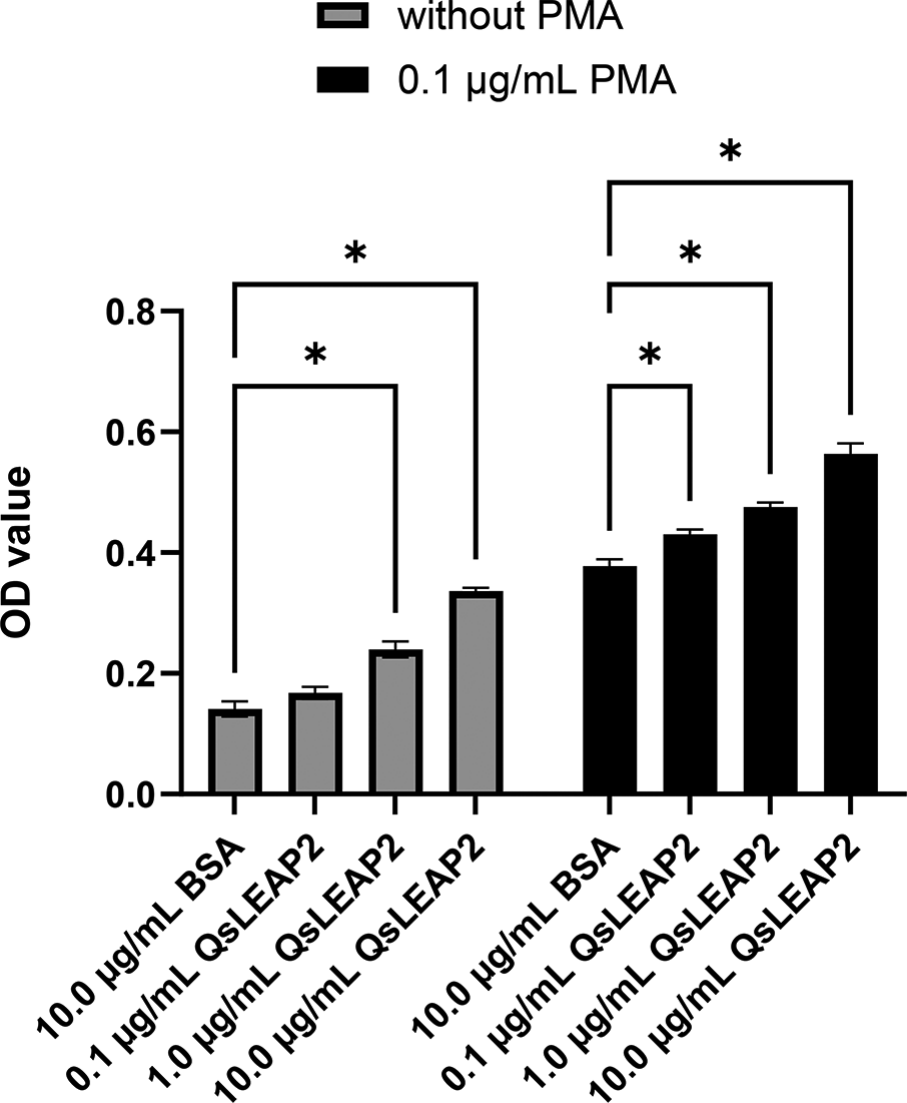
The impact of QsLEAP2 on the respiratory burst of RAW264.7 cells. The respiratory burst was quantified by assessing the optical density at a wavelength of 620 nm. Results are presented as the mean ± SEM, with *n* = 4. *P < 0.05

Following treatment with QsLEAP2, it was observed that the respiratory burst of RAW264.7 cells increased in accordance with the concentration of QsLEAP2. The respiratory burst of RAW264.7 cells treated with 10.0 µg/mL QsLEAP2 was observed to be 2.39-fold that of the BSA control. Furthermore, it was observed that the co-treatment of RAW264.7 cells with QsLEAP2 and PMA resulted in a significant enhancement of respiratory burst. The concentration of QsLEAP2 appeared to be a determining factor in this process, with higher concentrations leading to more pronounced effects. The co-treatment of RAW264.7 cells with 10.0 µg/mL of QsLEAP2 and PMA resulted in a respiratory burst that was 1.49-fold greater than that observed in the PMA and BSA co-treatment (Fig. 7).

### In Silico analysis of the interaction between QsLEAP2 and QsMOSPD2 proteins

**Fig. 8:**
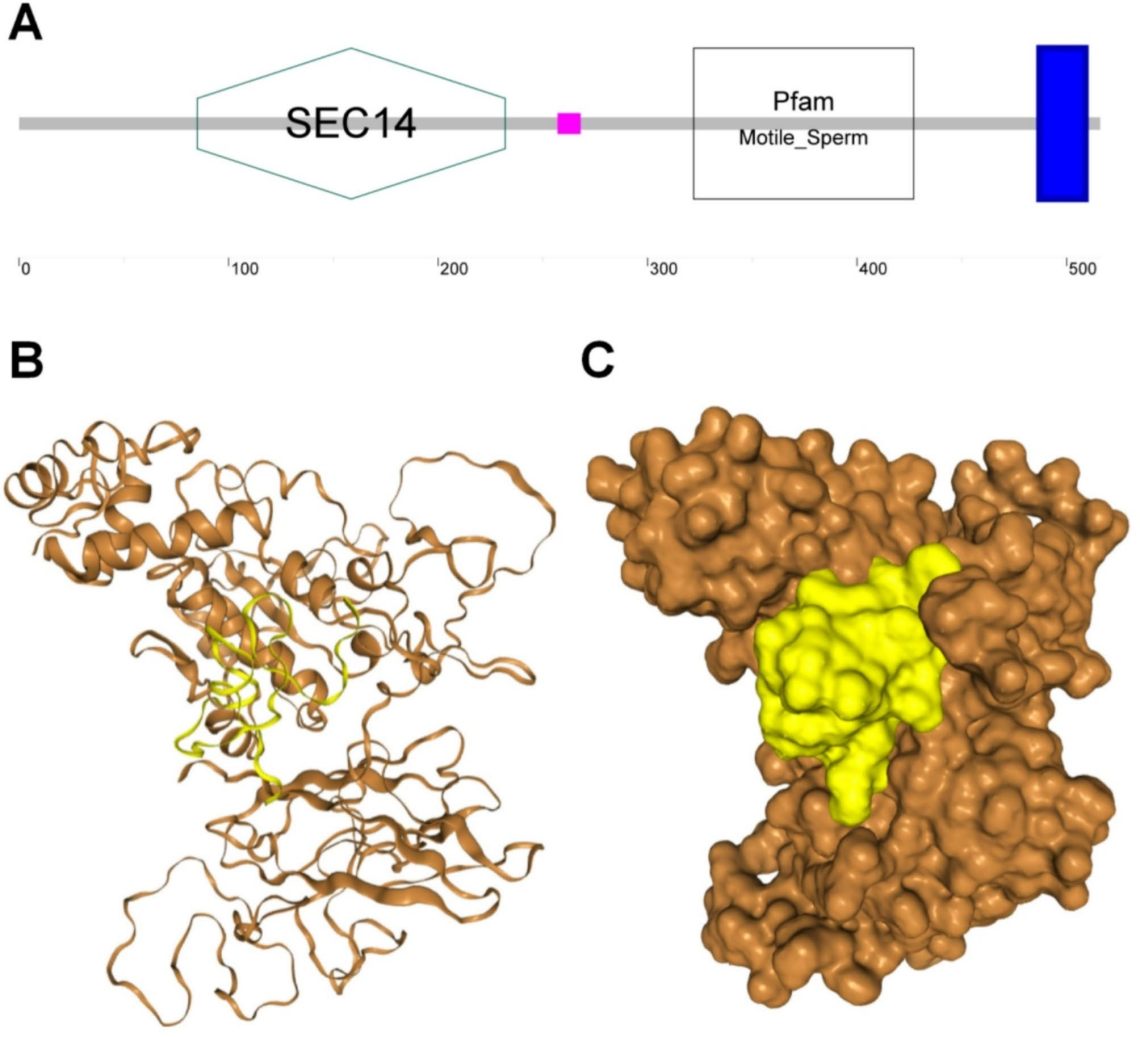
Molecular docking of QsLEAP2 and QsMOSPD2 conducted using HDOCK. (**A**) Domain architecture of QsMOSPD2 protein. The blue area represents the transmembrane region of QsMOSPD2. (**B**-**C**) In silico protein–protein docking. The interaction between QsLEAP2 and QsMOSPD2 is represented in the model using both cartoon and surface versions

LEAP2 regulates macrophage activity in fish via motile sperm domain-containing protein 2 (MOSPD2) [14]. The interaction between QsLEAP2 and QsMOSPD2 was examined using docking simulations on the HDOCK server, resulting in a model with a top binding score of -332.01 and a confidence score of 0.9744 (Fig. 8). QsLEAP2 was predicted to primarily bind to amino acid residues within the SCE14 and Mptile_Sperm domains of QsMOSPD2 (Supplementary Table 1), similar to binding within the groove formed by these two domains (Fig. 8).

## Discussion

LEAP2 is a small cysteine-rich cationic AMP crucial for innate immune responses [15]. In this study, we identified a LEAP2 homologue in Chinese spiny frogs. The predicted structure of QsLEAP2 included a signal peptide, a prodomain, and a mature peptide, which contained four conserved cysteine residues, as seen in the African clawed frog [12] and the Chong’an moustache toad [11]. These cysteine residues form two disulfide bonds essential for the peptide’s secondary structure [7]. Our structural model predictions align with this, showing that LEAP2 has a compact central core, while the N- and C-termini remain disordered. The core is stabilised by two intramolecular disulfide bonds and hydrogen bonds [7].

LEAP2 is a critical AMP in the host’s innate immune response, particularly in combating bacterial infections [16]. *QsLEAP2* exhibited the highest expression in the liver, with significant expression also in the spleen and kidneys. After infection with *A. hydrophila*, *QsLEAP2* expression was significantly upregulated in the liver, spleen, and kidneys. In the Chong’an moustache toad, *LEAP2* is highly expressed in the kidney and liver, with upregulation after *A. hydrophila* infection [11]. Similarly, in the golden pompano (*Trachinotus ovatus*), *LEAP2* is predominantly expressed in the liver, spleen, and kidneys with significant upregulation in response to bacterial infections such as *Edwardsiella tarda* and *Streptococcus agalactiae* [17].

The antibacterial properties of LEAP2 have been studied in various vertebrates [10, 15, 18–20]. For instance, LEAP2 from Chong’an moustache toad shows significant activity against both Gram-positive and Gram-negative bacteria [11]. In our study, the QsLEAP2 mature peptide exhibited strong antibacterial activity against *S. flexneri* and *V. alginolyticus*, followed by *S. enterica*, *S. aureus*, and *S. warneri*, indicating its broad-spectrum activity. AMP mechanisms often involve disrupting bacterial membranes [21] and LEAP2 increases membrane permeability, causing leakage of intracellular materials [11, 22]. This mechanism is similar to that of other AMPs, such as cathelicidin and esculentin [23, 24]. Additionally, LEAP2 can hydrolyse DNA, enhancing its antibacterial effects [11]. In our study, QsLEAP2 exhibited antibacterial activity by disrupting bacterial cell membranes and hydrolysing DNA, a mode of action that may reduce the development of resistance, a growing concern in bacterial infection treatment [25].

The difference between the MIC of QsLEAP2 against *S. flexneri* (3.125 µg/mL) and its concentration-dependent LDH release (significant at 100 µg/mL) indicates its multi-modal antibacterial action. At the MIC, QsLEAP2 likely inhibits growth through non-lytic mechanisms, such as membrane destabilisation or metabolic disruption, without causing immediate cell lysis. This is consistent with the functioning of other AMPs, which exert bacteriostatic effects at low concentrations and bactericidal activity at higher doses [1, 21]. The significant LDH release at 100 µg/mL suggests that membrane disruption becomes dominant, leading to cell lysis. This biphasic action—growth inhibition at MIC and lytic activity at higher doses—is typical of multiple AMPs such as cathelicidins and defensins [21, 22, 25].

LEAP2 has immunomodulatory properties in vertebrates [10, 14, 26], but its effects in amphibians remain unexplored. Previous studies show that vertebrate LEAP2 facilitates macrophage migration [10, 14]. Consistent with these findings, our study demonstrated that QsLEAP2 significantly enhanced macrophage migration, with higher concentrations increasing the number of migrating cells. Efficient macrophage migration is essential for infection control and healing during infectious or inflammatory conditions [27]. Consequently, LEAP2 may also augment the host immune response by enhancing macrophage migration. A previous study showed that pretreatment with GzLEAP-2B and GzLEAP-2 C from *Glyptothorax zanaensis* significantly enhances macrophage phagocytosis [28]. Consistent with this, our study revealed that QsLEAP2 significantly enhanced the phagocytic activity of RAW264.7 cells. QsLEAP2 also significantly increased the respiratory burst in these cells. Macrophages eliminate bacteria through phagocytosis, producing ROS and RNS, which contribute to respiratory bursts [29]. In fish, LEAP2 regulates macrophage activity via MOSPD2 [14]. In this study, we used the HDOCK server to simulate interactions between QsLEAP2 and QsMOSPD2, revealing a model where LEAP2 binds between the SCE14 and Mptile_Sperm domains of QsMOSPD2.

The multifunctional role of QsLEAP2 in Chinese spiny frog underscores the broader potential of AMPs as innovative solutions to combat antibiotic resistance and manage infectious diseases. Unlike conventional antibiotics, which often target specific bacterial pathways (e.g., cell wall synthesis or protein translation) and face rapid resistance evolution, AMPs exhibit a unique effector mechanism. Their cationic nature enables direct interaction with negatively charged bacterial membranes, leading to pore formation, membrane disruption, and the leakage of intracellular contents—a process less prone to resistance development due to the conserved physicochemical properties of microbial membranes [1, 21]. Moreover, AMPs such as QsLEAP2 often employ multi-modal strategies, such as hydrolysing bacterial DNA, thereby further complicating pathogen adaptation. This dual mechanism (membrane disruption and nucleic acid degradation) observed in QsLEAP2 exemplifies how AMPs bypass traditional resistance pathways linked to single-target antibiotics [2, 25].

Another critical advantage of AMPs lies in their immunomodulatory functions, synergising with their direct antimicrobial effects. While antibiotics solely target pathogens, AMPs actively enhance host immunity. For instance, QsLEAP2 not only killed *S. flexneri* but also stimulated macrophage chemotaxis, phagocytosis, and respiratory bursts. Such dual functionality amplifies the ability of the host to clear infections while minimising collateral damage to commensal microbiota, a common drawback of broad-spectrum antibiotics [4, 21]. This immunostimulatory role is particularly valuable in managing chronic or recurrent infections, where immune cell recruitment and activity are critical [5, 26].

The selective antibacterial activity of QsLEAP2 (e.g., efficacy against *S. flexneri* but not *E. coli*) highlights another strength of AMPs: pathogen specificity. Unlike indiscriminate antibiotics, AMPs can be engineered or naturally optimised to target priority pathogens while sparing beneficial microbes, thereby reducing dysbiosis risks [3, 25]. Furthermore, AMPs (such as QsLEAP2) are amenable to combinatorial therapies. For example, co-administration with conventional antibiotics could lower required doses, thereby mitigating toxicity and resistance pressures [25]. Advances in peptide stabilisation (e.g., cyclisation or D-amino acid substitution) and delivery systems (e.g., nanoparticles or liposomes) are addressing historical challenges such as proteolytic degradation and bioavailability, accelerating their clinical translation [3, 21].

While challenges remain (including production costs and potential cytotoxicity) the unique attributes of AMPs position them as next-generation therapeutics. QsLEAP2 exemplifies this promise, combining membrane disruption, DNA hydrolysis, and macrophage activation into a single peptide. Its interaction with QsMOSPD2 further suggests receptor-mediated immunomodulation, a feature exploitable for targeted immune therapies. As antibiotic pipelines stagnate, AMPs offer a versatile, evolutionarily refined alternative to address the global antimicrobial resistance crisis [1, 25].

## Conclusion

In conclusion, we identified and characterised a LEAP2 homologue from the Chinese spiny frog, designated QsLEAP2, which exhibited significant antibacterial activity against various bacterial species. QsLEAP2 compromised bacterial membrane integrity and hydrolysed bacterial genomic DNA, exerting bactericidal effects. Furthermore, QsLEAP2 induced chemotaxis in RAW264.7 cells, enhancing phagocytic activity and respiratory bursts. Simulations revealed interactions between QsLEAP2 and QsMOSPD2. Although QsLEAP2 exhibits promising immunomodulatory activity in a murine macrophage model, its role in amphibian host defence remains speculative. The results of study underscore the necessity for species-specific validation to further elucidate AMP-driven immunity in non-mammalian vertebrates and to evaluate the therapeutic potential of QsLEAP2 in amphibians.

## Methods

### Experimental animals

Experimental Chinese spiny frogs were obtained from Longquan Fuxi Agricultural Development Co., LTD, Longquan City, China. The animals, weighing 150 ± 10 g, were maintained in freshwater at a temperature of 18–20 ℃. Their diet consisted of a commercial formulation, administered twice daily. Prior to the commencement of the experiments, the frogs were acclimated to laboratory conditions for two weeks.

### Molecular characterisation of QsLEAP2 cDNA

The *QsLEAP2* cDNA sequence was extracted from the liver transcriptome (PRJNA1012438), and the molecular mass and isoelectric point of the encoded protein were calculated using ProtParam. SignalP 5.0 was employed to predict the cleavage sites for the signal peptides. AlphaFold2 was used to model the peptides [30]. ClustalW was used to analyse multiple alignments. The MEGA X software was used to construct a phylogenetic tree.

### Constitutive and infection-induced expression of QsLEAP2

The liver, spleen, kidney, lungs, gut, skin, heart, and muscles were obtained from four healthy Chinese spiny frogs for constitutive expression analysis. The *A. hydrophila* challenge was conducted in accordance with a previously described method [23]. In brief, *A. hydrophila* (1.0 × 10^4^ CFU; 1/10 of the LD50) was intraperitoneally (ip) injected into Chinese spiny frogs (*n* = 8). Sterile saline solution was injected in the control group (*n* = 4). Tissues (liver, spleen, kidney, gut, and lungs) were collected 12 h and 24 h post-infection. Four samples were collected for each tissue and stored at -80 °C. All frogs were anaesthetised with 0.1 g/L tricaine methanesulphonate (MS-222) prior to injection and dissection.

### Real-time quantitative PCR

Total RNA was extracted from each sample using TRIzol reagent (Beyotime), and cDNA was synthesised using the PrimeScript RT kit with gDNA Eraser (TaKaRa). Subsequently, the BeyoFast™ SYBR Green qPCR Mix (2X) (Beyotime) and a real-time PCR detection system (CFX96; Bio-Rad) were used to perform qPCR to detect the cycle threshold of *QsLEAP2* and *Qs18S rRNA*. The primers used in this study are listed in Table 2. The relative expression levels of *QsLEAP2* and *Qs18S rRNA* were calculated using the 2^−ΔΔCt^ method [31].

**Table 2:** Oligonucleotide primers

Gene	Primers	Sequence (5′-3′)
*QsLEAP2*	LEAP2-t(+)	TAGGACAAAGGAGCGACTGG
	LEAP2-t(-)	GGAGGAAGGAGCAGTGTCTT
*Qs18S rRNA*	18 S rRNA-t(+)	TTAGAGGGACAAGTGGCGTT
	18 S rRNA-t(-)	TGCAATCCCCGATCCCTATC

### Antibacterial assay

The mature peptide of QsLEAP2 (MTPFWRAVGNKPVGAYCQQGLECTTKVCRRRHCSFLQHNWS) was chemically synthesised with a purity of over 95% (SynPeptide, Shanghai, China). The antibacterial activity of the peptide was evaluated against a panel of bacterial strains including *S. aureus*, *S. saprophyticus*, *L. monocytogenes*, *S. warneri*, *S. flexneri*, *V. alginolyticus*, *S. enterica*, *A. hydrophila*, *P. mirabilis*, *P. aeruginosa*, and *E. coli*. The MIC of the QsLEAP2 mature peptide was determined using a modified two-fold microdilution method as described previously [32]. Twofold serial dilutions of the peptide were prepared in sterile 96-well plates, ranging from 1000 to 31.25 µg/mL. We added 10 µL of the diluted peptide in each well, followed by the addition of 90 µL of mid-logarithmic phase bacterial suspension adjusted to a final density of 1.0 × 10⁵ CFU/mL. After 24 h of incubation at optimal growth temperatures, the MIC was determined by measuring optical density at 600 nm. All assays were conducted in triplicates with independent biological repeats.

### Lactate dehydrogenase (LDH) release assay

Bacterial membrane disruption was assessed using the LDH Release Assay Kit (Beyotime) with protocol adaptations. *S. flexneri* suspensions (1 × 10⁹ CFU/mL in PBS) were exposed to gradient concentrations of QsLEAP2 (25, 50, or 100 µg/mL) for 2 h at 37 °C. After the treatment, we centrifuged the samples at 8,000 × g for 2 min and analysed the supernatants in triplicates using 96-well plate assays: 120 µL supernatant mixed with 60 µL LDH detection working solution (30 min incubation at room temperature), with absorbance measured at 490 nm using a microplate reader.

### DNA hydrolysis assay

Hydrolysis of genomic DNA from *S. flexneri* was assayed using a previously described methodology [33]. The genomic DNA of *S. flexneri* was extracted using an Ezup Column Bacteria Genomic DNA Purification Kit (Sangon), according to the manufacturer’s instructions. We combined QsLEAP2 at concentrations of 25, 50, or 100 µg/mL with 800 ng of genomic DNA. Following a 30-min incubation period, a 6× loading buffer was added to the mixture. Subsequently, the reaction solution was subjected to electrophoresis on a 1.0% agarose gel using a 0.5× TAE buffer system. DNA hydrolysis assays were performed in triplicates and repeated at least once.

### Chemotaxis assay

To evaluate the QsLEAP2 macrophage recruitment capacity, we used Transwell chambers in accordance with established protocols [24]. Chemotaxis assays were conducted in a constant humidity incubator maintained at 37 °C with 5% CO_2_, using 24-well Transwell chambers (Corning, pore size 5 μm). RAW 264.7 cells (6 × 10^4^ cells) were seeded in the upper chamber, while the lower chamber was supplemented with DMEM medium containing varying concentrations of QsLEAP2 (0, 0.1, 1.0, and 10.0 µg/mL) for a fixed incubation period of 24 h. The rate of cell migration was quantified as follows: (1) After Wright-Giemsa staining, five non-overlapping fields of view were randomly selected under a 400x microscope; (2) Validation was performed through manual counting and using the ImageJ software (v1.53t, Particle Analysis Module), counting only cells that had completely traversed the membrane; and (3) The migration rate was calculated using the following formula: the number of migrated cells in the treatment group divided by the number of cells in the control group. The final data represent the average of three independent experiments.

### Phagocytosis assay

To assess the impact of QsLEAP2 on the phagocytic activity of RAW264.7 cells, a flow cytometric assay using FITC-dextran uptake was employed, as described previously [34]. RAW264.7 cells (5 × 10⁵/well) were preconditioned in DMEM supplemented with 10% heat-inactivated FBS during a 12-h exposure to QsLEAP2 (0, 0.1, 1.0, or 10.0 µg/mL) to simulate physiological opsonisation. Following treatment, cells were washed three times with ice-cold PBS (centrifugation: 300×g, 5 min per cycle) to remove peptides, then incubated with 1 mg/mL FITC-dextran in serum-free RPMI-1640 at 37 °C/5% CO₂ for 30 min. Surface-adherent fluorescence was quenched using 0.4% trypan blue (*p*H 4.4) for 1 min prior to analysis. Flow cytometry was performed on a Beckman CytoFLEX LX instrument, employing a three-step gating strategy: (1) FSC-A/SSC-A thresholding (50,000 events) to exclude debris; (2) FSC-H/FSC-A discrimination for single-cell selection; (3) FITC⁺ detection (488/525 nm) with compensation controls. The mean fluorescence intensity (MFI) was reported as a fold-change in comparison to the BSA-treated control group, which was arbitrarily set to a value of 100. Phagocytosis assays were performed in triplicate and repeated at least once.

### Respiratory burst assay

To assess the impact of QsLEAP2 on the production of reactive oxygen species (ROS) in RAW264.7 cells, the respiratory burst assay was conducted. This involved quantifying the intracellular concentration of O^2−^ through the Nitro Blue Tetrazolium (NBT) reduction test, following the methodology outlined in a previous study [26]. The cells were pretreated with QsLEAP2 (0, 0.1, 1.0, 10.0 µg/mL) for 12 h. Next, they were treated with or without PMA (0.1 µg/mL). The cells were incubated with NBT (1 mg/mL) at 24 °C for 1 h and the reaction was halted with methanol. The cells were purified and dried. Formazan was prepared in a mixture of KOH and DMSO, and its optical density was measured at 620 nm. Respiratory burst assay were performed in triplicate and repeated at least once.

### In Silico protein–protein Docking

In order to predict the interaction between QsLEAP2 and QsMOSPD2, the HDOCK server was employed [35]. The *QsMOSPD2* cDNA was obtained from the liver transcriptome (PRJNA1012438). The domain of QsMOSPD2 was predicted using the SMART software. Structural modelling of QsMOSPD2 was performed using the AlphaFold2 server [30]. QsLEAP2 docking with QsMOSPD2 was performed using the HDOCK server [35]. The default parameters were used for docking.

### Statistical analysis

Data are shown as means ± SEM. The results were analysed using a one-way analysis of variance (ANOVA) with SPSS 13.0. *P* values < 0.05 were deemed statistically significant.

## Electronic supplementary material

Below is the link to the electronic supplementary material.

Supplementary Material 1

Supplementary Material 2

## Data Availability

The QsLEAP2 cDNA sequence was submitted to GenBank under accession number PQ528275. The datasets used and/or analysed during the current study are available from the corresponding author on reasonable request.
