# Supplementary material for: Identification and characterisation of LEAP2 from Chinese spiny frogs (Quasipaa spinosa) with antimicrobial and macrophage activation properties
Source: BMC Vet Res. 2025 Mar 13;21:163. doi: 10.1186/s12917-025-04617-y (PMC11905587; doi:10.1186/s12917-025-04617-y)
Supplement: Supplementary file 1 — Supplementary Material 1 [file 12917_2025_4617_MOESM1_ESM.docx]

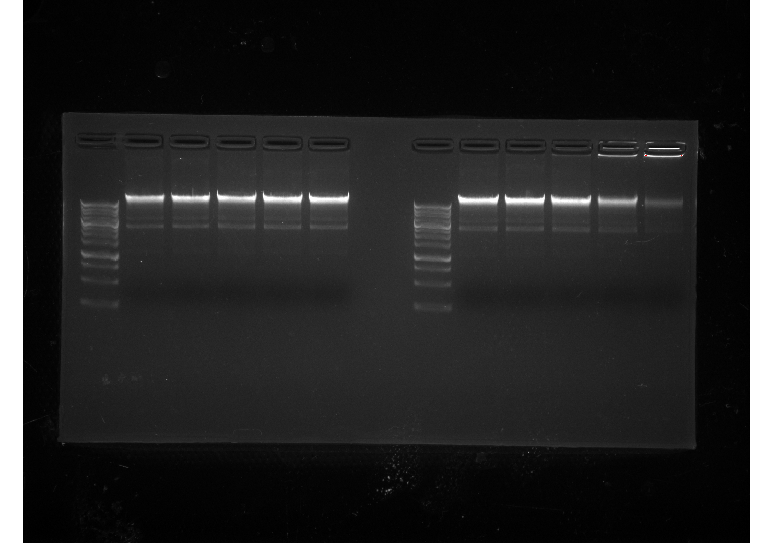


**Fig. 4.** (B) The hydrolytic activity of QsLEAP2 on the bacterial genomic DNA was assessed through electrophoresis. BSA was employed as a negative control.
