# Supplementary material for: Identification and characterisation of LEAP2 from Chinese spiny frogs (Quasipaa spinosa) with antimicrobial and macrophage activation properties
Source: BMC Vet Res. 2025 Mar 13;21:163. doi: 10.1186/s12917-025-04617-y (PMC11905587; doi:10.1186/s12917-025-04617-y)
Supplement: Supplementary file 2 — Supplementary Material 2 [file 12917_2025_4617_MOESM2_ESM.docx]

**Supplementary Table 1.**

QsMOSPD2-QsLEAP2 interface residue pairs

| QsMOSPD2 interface residues | QsLEAP2  interface residues | QsMOSPD2-QsLEAP2 interface residue pairs |
| --- | --- | --- |
| ASN 74 | MET 1 | 74 - 31 |
| GLU 78 | THR 2 | 78 - 30 |
| VAL 125 | PRO 3 | 78 - 31 |
| PHE 127 | TRP 5 | 78 - 32 |
| ARG 131 | ARG 6 | 125 - 29 |
| PHE 162 | VAL 13 | 125 - 40 |
| CYS 166 | GLY 14 | 127 - 29 |
| ILE 169 | ALA 15 | 127 - 30 |
| TYR 170 | TYR 16 | 127 - 39 |
| ARG 173 | ARG 29 | 127 - 40 |
| TYR 174 | ARG 30 | 131 - 30 |
| SER 176 | ARG 31 | 162 - 5 |
| LYS 177 | HIS 32 | 162 - 13 |
| PRO 200 | CYS 33 | 162 - 35 |
| ASN 204 | SER 34 | 166 - 13 |
| MET 205 | PHE 35 | 169 - 14 |
| PHE 297 | LEU 36 | 170 - 13 |
| LYS 298 | GLN 37 | 170 - 14 |
| HIS 299 | HIS 38 | 170 - 15 |
| SER 302 | ASN 39 | 170 - 32 |
| ASP 306 | TRP 40 | 170 - 33 |
| LEU 323 |  | 170 - 34 |
| HIS 324 |  | 173 - 14 |
| ILE 325 |  | 173 - 15 |
| SER 326 |  | 173 - 16 |
| ALA 328 |  | 173 - 32 |
| THR 413 |  | 174 - 16 |
| GLN 471 |  | 174 - 32 |
| LEU 473 |  | 176 - 16 |
| PHE 505 |  | 177 - 16 |
| LEU 508 |  | 177 - 31 |
| TYR 509 |  | 177 - 32 |
| MET 510 |  | 200 - 6 |
| LEU 511 |  | 204 - 5 |
| ASP 513 |  | 204 - 6 |
|  |  | 205 - 14 |
|  |  | 205 - 15 |
|  |  | 205 - 16 |
|  |  | 297 - 40 |
| QsMOSPD2 interface residues | QsLEAP2  interface residues | QsMOSPD2-QsLEAP2 interface residue pairs |
|  |  | 298 - 39 |
|  |  | 298 - 40 |
|  |  | 299 - 38 |
|  |  | 299 - 39 |
|  |  | 299 - 40 |
|  |  | 302 - 39 |
|  |  | 306 - 37 |
|  |  | 306 - 39 |
|  |  | 323 - 37 |
|  |  | 324 - 37 |
|  |  | 325 - 35 |
|  |  | 326 - 36 |
|  |  | 328 - 2 |
|  |  | 413 - 1 |
|  |  | 471 - 2 |
|  |  | 471 - 3 |
|  |  | 471 - 6 |
|  |  | 473 - 1 |
|  |  | 505 - 5 |
|  |  | 505 - 35 |
|  |  | 508 - 2 |
|  |  | 509 - 2 |
|  |  | 510 - 2 |
|  |  | 510 - 3 |
|  |  | 510 - 5 |
|  |  | 511 - 5 |
|  |  | 513 - 6 |

SCE14 domain: 85aa-232aa; Mptile_Sperm domain: 322aa-437aa
